# Glancing at the past and course-setting for the future: lessons from the last decade of research on medication abortion in high-income countries

**DOI:** 10.1186/s12978-021-01081-3

**Published:** 2021-02-08

**Authors:** Annik M. Sorhaindo

**Affiliations:** Independent Consultant in Reproductive and Sexual Health, Calle Agrarismo 65, Colonia Escandón II, Delegación Miguel Hidalgo, CP11800 Mexico City, Mexico

**Keywords:** Medication abortion, Quality assessment, Clinical research, Service delivery, Demedicalization

## Abstract

**Objective:**

Although medication abortion has become more common in high-income countries, the procedure has not yet met early expectations for widening access to abortion. High-quality evidence can serve as a catalyst for changes in policy and practice. To direct research priorities, it is important to understand where quality evidence is concentrated and where gaps remain. High-income countries have developed a body of evidence that may have implications for the future of medication abortion. This literature review assesses the characteristics and quality of published studies on medication abortion conducted in the last 10 years in high-income countries and indicates future areas for research to advance policy and practice, and broaden access.

**Study design:**

A structured search for literature resulted in 207 included studies. A framework based upon the World Health Organization definition of sub-tasks for medication abortion was developed to categorize research by recognized stages of the medication abortion process. Using an iterative and inductive approach, additional sub-themes were created under each of these categories. Established quality assessment frameworks were drawn upon to gauge the internal and external validity of the included research.

**Results:**

Studies in the US and the UK have dominated research on MA in high-income countries. The political and social contexts of these countries will have shaped of this body of research. The past decade of research has focused largely on clinical aspects of medication abortion.

**Conclusion:**

Researchers should consider refocusing energies toward testing service delivery approaches demonstrating promise and prioritizing research that has broader generalizability and relevance outside of narrow clinical contexts.

**Plain English summary:**

Although medication abortion is more commonly available worldwide, it is not being used as often as people thought it would be, particularly in high income countries. In order to encourage changes in policy and practice that would allow greater use, we need good quality evidence. If we can understand where we do not have enough research and where we have good amounts of research, we can determine where to invest energies in further studies. Many high-income countries have produced research on medication abortion that could influence policy and practice in similarly resourced contexts.

I conducted a literature review to be able to understand the type and quality of research on medication abortion conducted in high-income countries in the past 10 years. I conducted the review in an organized way to make sure that the papers reviewed discussed studies that I thought would be important for answering this question.

The literature review found 207 papers. Each of these papers were reviewed and organized them by theme. I also used existing methods to determinine the quality of each study.

Most of the research came from the US and the UK. Furthermore, most of the research conducted in the past 10 years was focused on clinical studies of medication abortion.

In future studies, researchers should focus more on new ways of providing medication abortion to women that offers greater access. Also, the studies should be designed so that the results have meaning for a broader group of people or situations beyond where the study was done.

## Introduction

Medication abortion (MA) has changed the landscape of abortion provision. The last decade experienced growth in the proportion of all abortions conducted using MA. In high-income countries, MA accounts for at least half of all abortions. However, this varies by country; with the highest proportions in countries such as Finland and Sweden, and lower proportions in countries such as Canada, Italy and Belguim [1]. In the United States (US), MA accounts for approximately 40 percent of all abortions, and is the most common method of early abortion [2].

Despite current availability and access, MA has yet to meet early expectations of widespread, demedicalized use. Even in high-income countries with well-functioning health systems and fewer legal contraints, barriers to broad use of MA persist [3–5]. International and national variations in regulatory, social and political environments have limited the capacity for scaling access and introducing innovative approaches to service delivery that would allow women more control over their abortion experiences [3]. Further expansion of MA has the potential to greatly improve the experiences of women seeking abortion by increasing the options available to them, both in terms of abortion methods and means of accessing services [6].

Demedicalization of MA is one mechanism for simplifying and sustaining access [7, 8]. High-income countries have developed a significant body of evidence on MA. Leveraging and sharing evidence across contexts with similarly well-resourced health care systems has the potential to influence MA policy and practice. To inform and shape future research priorities, it is important to understand topic areas where quality evidence is concentrated and where gaps remain.

Literature reviews can be used to assess the quantity and types of studies that currently exist on a specific topic in preparation for the development of an agenda for additional research, both primary research and synthese of evidence, in order to determine where there is sufficient information and where more evidence is needed [9, 10]. This review maps the landscape of the last decade of research on MA conducted in high-income countries and considers what additional research is needed to enable women in these contexts to have simpler access to abortion with fewer barriers.

## Material and methods

This review was undertaken in three steps: (1) structured search and identification of relevant research studies, (2) categorization of selected studies by MA thematic area, (3) and assessment of the methodological quality of the research.

To identify studies on MA conducted in high-income countries, and published in 2007–2017, a search of bibliographic databases PubMed, POPLINE, Embase, Global Health, and Web of Science (search strategy available upon request) was conducted. World Bank classifications were used to limit the bibliographic search high-income countries [11]. Inclusion and exclusion criteria were applied to the list of titles and abstracts resulting from the bibliographic search to identify full-texts for further analysis. To be *included,* the publication had to: (1) focus on an aspect of MA, (2) be based on data collected in a high-income country and (3) be written in English. Additional exclusion criteria were applied to full texts, and the following types of articles were excluded: conference and poster abstracts; editorials, corrections, case reports, reviews of literature etc.; not primarily focused on medication abortion; focused on non-elective abortions (miscarriage, missed abortion, fetal anomaly or demise); not in English; focused on the mechanism of action for MA; and identifiably not from a high-income country. The remaining studies were included.

Each of the remaining studies were categorized by three major characteristics: study methodology, trimester of pregnancy among study subjects, and country of study. A framework based upon the World Health Organization (WHO) definition of sub-tasks for MA was developed to categorize research by recognized stages of the MA process: (1) assessing eligibility for MA, (2) administering the medications and managing the process, and (3) assessing completion of the procedure and need for follow-up [12].

Using an iterative and inductive approach, sub-themes were created under each of the categories to represent thematic areas emerging from the literature (Table 1). Any studies reporting on research using different techniques for determining eligibility for MA were included under eligibility assessment. Within the medication administration category, two sub-categories for clinical management and models of service delivery were developed. Research on assessing completion and the need for follow-up, included sub-categories on failure and adverse events, the use of β-hCG measurements and/or ultrasound to assess abortion completion, models of post-abortion follow-up, and post-MA contraception. For study themes that did not fit within the WHO sub-tasks for MA, two additional categories were created labeled “other clinical research” and “social science research.” Any of the included studies could be categorized into more than one study theme.

**Table 1:** Subcategories and definitions by WHO MA subtask

Category		Subcategory	Definition
WHO Subtask 1. Assessing eligibility		Eligibility assessment	Using different mechanisms, such as LMP and pelvic bimanual versus ultrasound to determine eligibility for early MA
WHO Subtask 2: Administering the medications and managing the process and common side-effects	Clinical management	Safety and efficacy	Testing different clinical innovations and regimens for MA
		Feasibility	Practicability of administration of mifepristone-misoprostol or misoprostol-only regimens on MA in various situations and contexts
		Management of side effects and complications	Self-administration of medication and self-management of pain, bleeding, expulsion of the products of conception, and self-identification of the need to seek formal healthcare for potential complications
	Models of service delivery	Facility-based models	Assessment of different models of facility-based provision of MA
		Information and counseling	Models of providing and receiving information on MA before undergoing the procedure
		Online and telemedicine provision	Provision or acquisition of MA pills and/or information about the procedure via website or via telemedicine, i.e. providers using telecommunications technology to interact with patients remotely
		Home use	Safety, effectiveness and experiences of administration of mifepristone-misoprostol or misoprostol-only regimens by an individual at home. This also includes partial self-administration
		Pharmacy provision	Documentation of sourcing of MA from pharmacists or pharmacies, regardless of the legal context
WHO Subtask 3: Assessing completion of the procedure and the need for further clinic-based follow-up		Failure and adverse events related to MA	Prevalence and characteristics of adverse events, including failure and need for surgical intervention, hospital admission, blood transfusion, emergency department treatment, intravenous antibiotics administration, infection, and death, as follow-on events from cases of self-administration of combined regimen and/or misoprostol-only induced abortions
		Post-abortion follow-up using β-hCG	Using serum hCG measurements for monitoring of abortion completion versus or in place of ultrasonography
		Models for post-abortion follow-up	Effectiveness of different types of MA service delivery follow-up options, e.g. home pregnancy test, checklists, bimanual or speculum examination by provider, telephone follow up etc. to assess completion
		Ultrasound	Techniques or technology associated with conducting and/or reading ultrasound as part of the MA process – only for completion
		Post-MA contraception	Take up, safety and acceptability of contraceptive methods after MA
Other clinical		Prevalence	Analyses of proportions of a population experiencing (aspects of) MA; can include subgroup analyses, and association and correlations with other factors
		MA in particular populations	Analyses of the safety, efficacy, acceptability and service delivery options for certain sub-groups of a given population
		Surgical vs. medical abortion	Comparison of surgical and MA regarding factors such as preference, access, acceptability, safety, efficacy etc
Social science		Knowledge, attitudes and practices	Assessing the awareness, views and behaviors of different populations regarding MA among women, partners, providers and relevant others
		Women's preferences and experiences with MA	Measure of preferences regarding MA among women who have used it
		Legal/policy context	Related to laws and policies governing MA
		Cost-effectiveness	Economic analyses of relative costs and outcomes of different aspects of the delivery and/or receipt of MA care // Documentation of the degree to which a specific aspect of MA is good value for the resources required

Established quality assessment frameworks were drawn upon to gauge the internal and external validity of the included research. Separate quality assessment frameworks were developed for quantitative studies and for qualitative studies. The rating scheme for quantitative studies was developed based on the US Preventive Services Task Force’s procedure manual, which provides guidance on assessing systematic reviews, case–control studies, randomized controlled trials (RCTs), and cohort studies, and the AXIS checklist was adapted to create a comparable rating approach for cross-sectional and observational studies [13, 14]. Assessment of internal validity considered how well a study adhered to key tenets for reducing risk of bias, for example, including criteria such as clarity of study aims and objectives, response rate and maintenance of comparable groups. The assessment of external validity considered whether the study was sufficiently robust based on study population, setting, sample size, sampling strategy and recruitment, refusal rate and loss-to-follow-up, to generalize the findings.

For qualitative studies, the rating scheme designed drew upon the principles of the Confidence in the Evidence from Reviews of Qualitative research (CerQual) approach to assessing confidence in findings [15]. Each included qualitative study was assessed against the following criteria: the appropriateness of qualitative methods, the research design, and the recruitment strategy to the aim of the research; consideration of the relationship between the researcher and particpants, and ethics; rigour of the data anlysis and whether the findings were supported by evidence. All studies received a rating of good, fair, or poor for internal and external validity.

As both the development of themes and quality assessments were conducted by one researcher, internal consistency checks were conducted regularly to increase the reliability of categorizations and ratings. Full texts were reviewed, considered and compared in multiple rounds before the results were finalized to minimize the risk of bias and inconsistency.

## Results

**Fig. 1:**
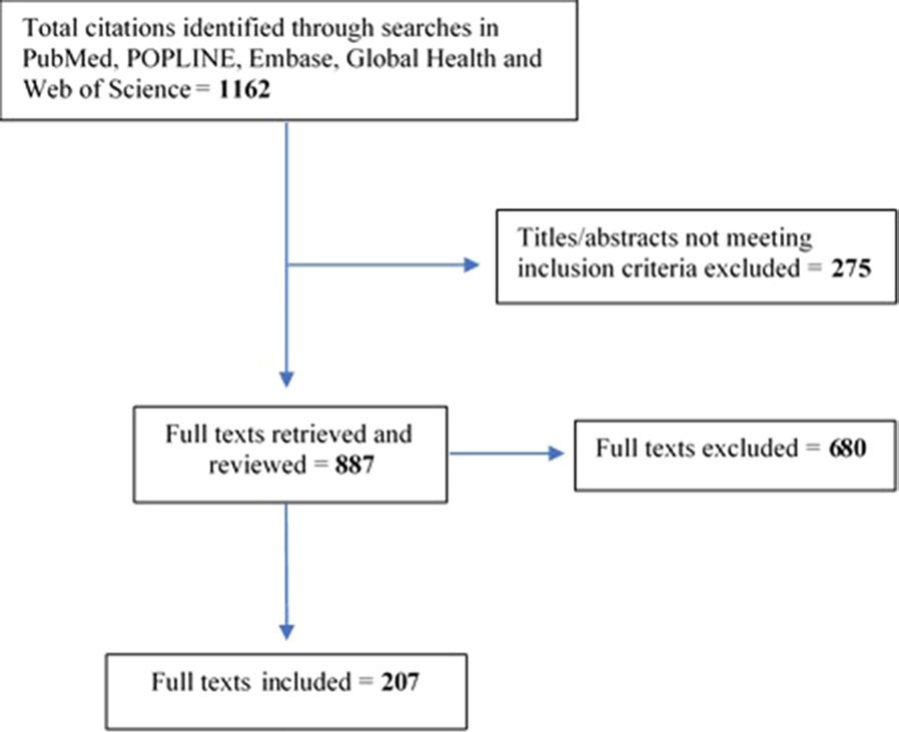
Study flow chart

This search produced 1162 records (Fig. 1). The titles and abstracts of all records were reviewed, and records not meeting inclusion criteria were excluded (n = 275) and full texts of relevant articles retrieved (n = 887). Of 887 full texts retrieved, 680 did not meet review criteria and were excluded. A final set of 207 papers were included in the literature review.

## Description and categorization of included studies

Study design and gestational age.

Table 2 provides a distribution of the included studies by study design and gestational age of pregnancies. All except 23 of the studies employed soley quantitative methods. The remaining studies used qualitative (n = 19) and mixed methods designs (n = 4). About two-third of the studies focused on MA in the first trimester of pregnancy.

**Table 2:** Distribution of studies by study design and gestational age of pregnancies

Categorization	Number of studies
Study design	
Cohort	101
Cross-sectional/Observational	48
Randomized Controlled Trial	30
Qualitative	19
Case–control	5
Mixed methods	4
Trimester	
First	137
Second	27
Third	16
Not stated	27
Total	207

### Location of studies

**Fig. 2:**
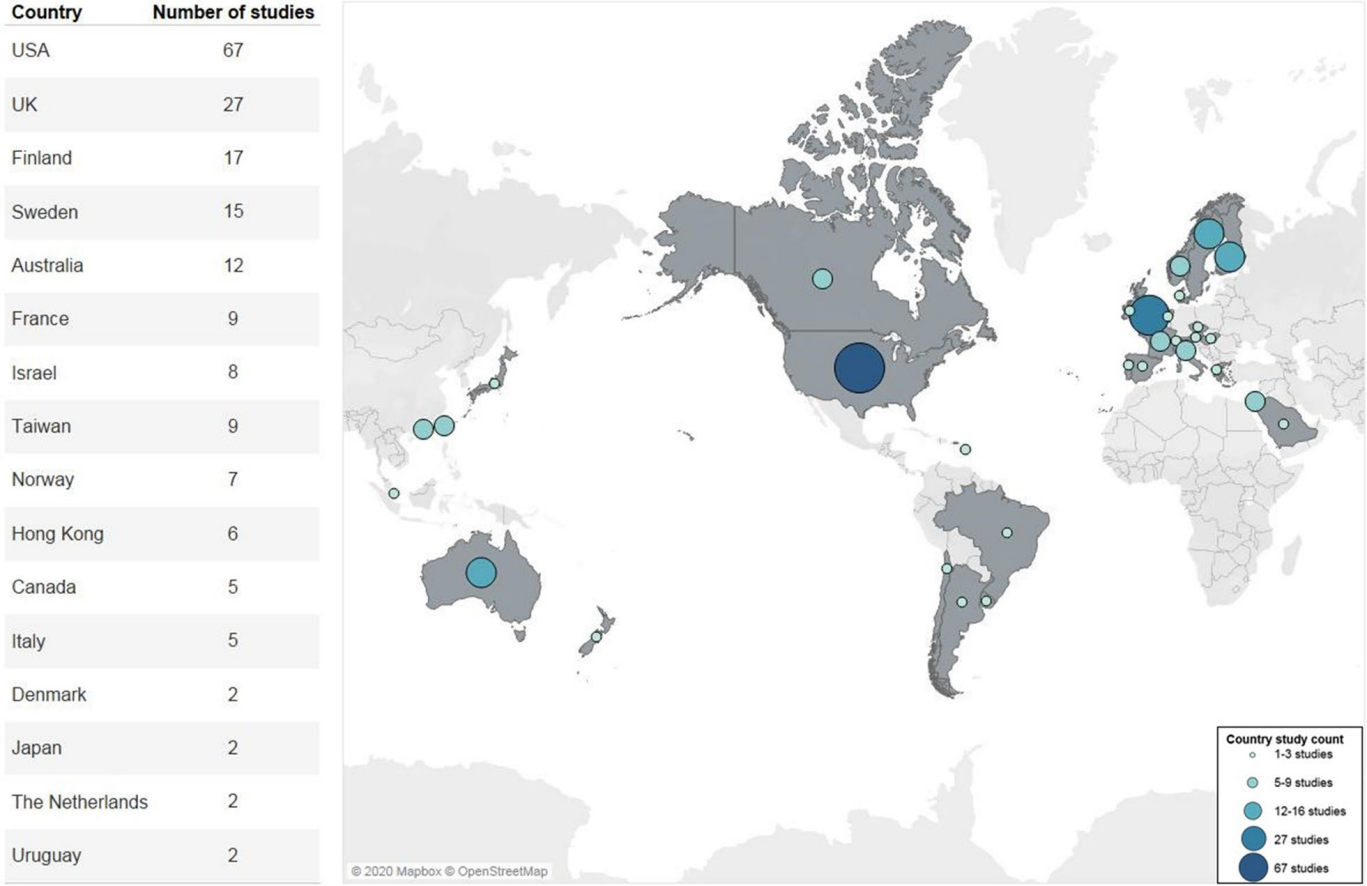
Countries represented in included studies

Included studies were conducted in 35 high-income countries. However, a majority of the studies were conducted in five only countries: USA (n = 67), UK (n = 27), Finland (n = 17), Sweden (n = 15) and Australia (n = 12) (Fig. 2).

Table shows only countries where two or more included studies took place. One included study was conducted in each of the following countries: Argentina, Austria, Brazil, Chile, Czech Republic, Greece, Hungary, Ireland, New Zealand, Portugal, Saudi Arabia, Singapore, Spain, Switzerland, and the Caribbean countries/territories of Anguilla, Antigua, Curacao, St. Martin/St. Maarten, and St. Kitts. Some studies covered more than one country; total may not equal 207.

### Study themes

**Fig. 3:**
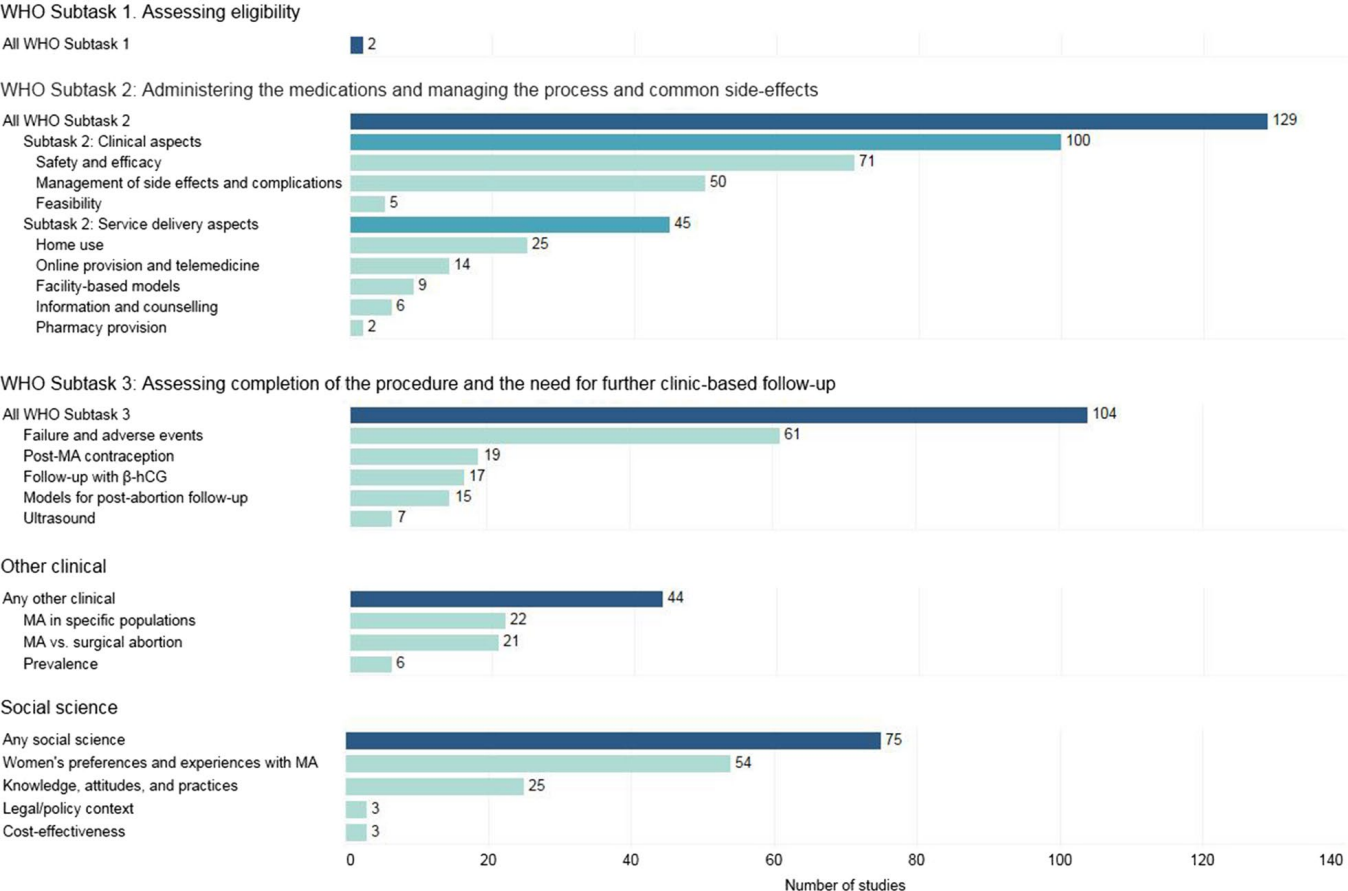
Number of included studies by topic area

Among the 207 included studies on MA conducted in high-income countries, only 2 focused on issues related to assessing women’s eligibility for MA. Nearly two-thirds (n = 129) of all studies focused on administering medications and managing the MA process and side effects. The bulk of these studies (n = 100) assessed the clinical aspects of MA and 45 researched service delivery aspects of MA. Among the 100 studies on clinical aspects of MA, around 70 percent focused on assessments of safety and efficacy and about half considered the management of side effects and complications. Only 5 of these studies documented research on the feasibility of administering MA. Of the 45 studies on service delivery aspects of MA, 25 evidenced some aspects of home use (Fig. 3).

One hundred and four studies focused on the assessment of completion of the MA procedure and need for further clinic-based follow-up. The bulk of these papers (n = 61) were studies on failure and adverse events. Only 7 of these papers studied ultrasound as a feature of assessing completion and follow-up (Fig. 3).

Over half of all the studies (n = 119) were classified as “other clinical” and/or “social science”. Among the social science studies, the greatest number documented women’s preferences and experiences of MA (n = 54). Only 6 papers discussed on the legal/policy context of MA (n = 3) or cost-effectiveness of MA (n = 3). Among “other clinical” studies most looked at MA in specific populations (n = 22) or issues related to MA versus surgical abortion (n = 21). Few studies in this category (n = 6) considered the prevalence of the use of MA (Fig. 3).

See definitions for WHO Subtasks and subcategories in Additional file 1.

### Quality of included studies

**Fig. 4:**
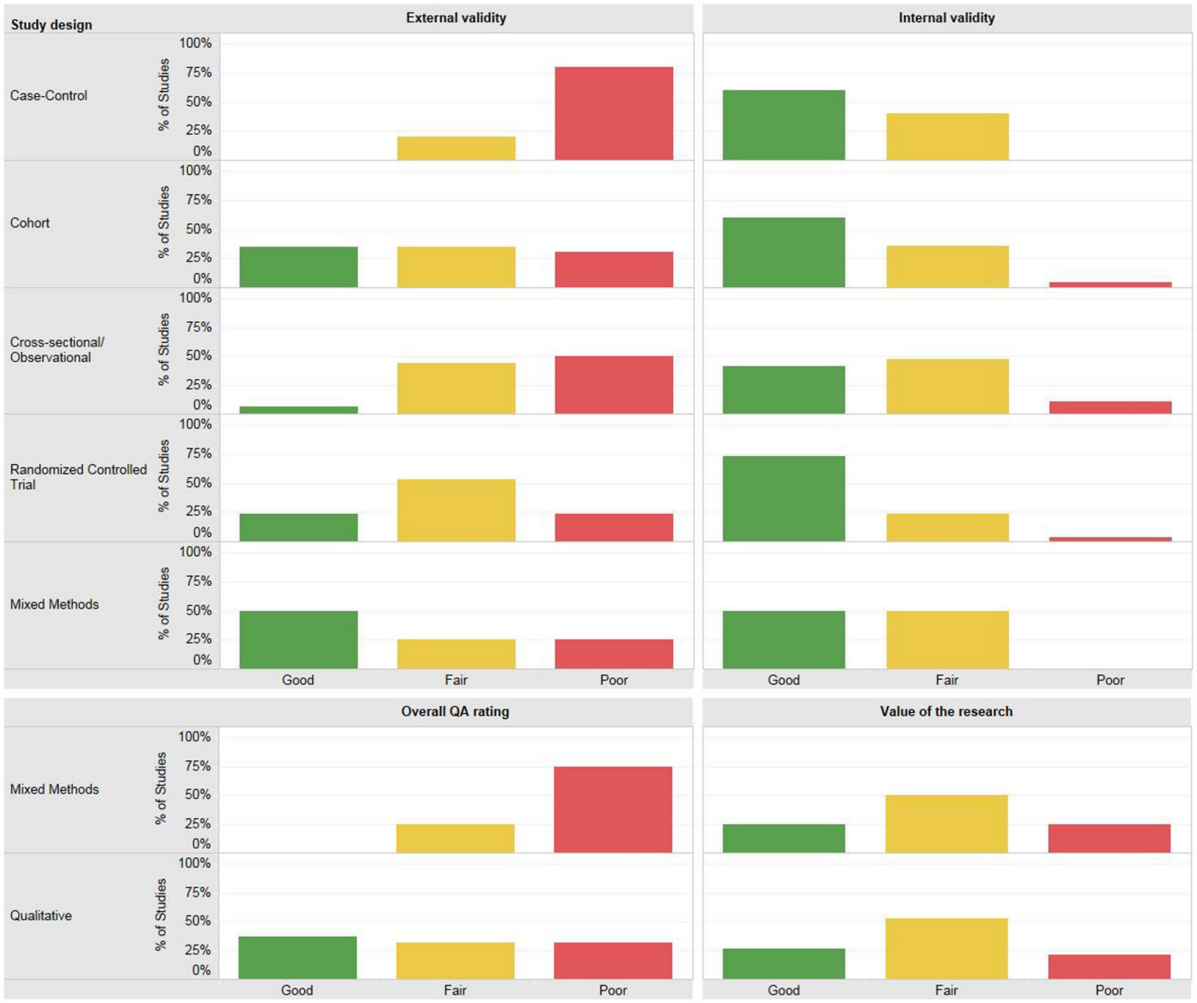
Quality assessment ratings by type of study design

The included quantitative studies tended to score either good or fair for internal validity, meaning that the risk of bias among these studies was relatively low. Alternatively, judgments of external validity were more moderate; tending more toward scores of fair or poor (Fig. 4).

As noted above, only 19 of the included studies used qualitative methods. Overall, the value of the qualitative research included in this analysis was judged to be mostly fair. This means that the authors gave moderate consideration to how their findings expanded understanding of the topic and provided modest discussion of the next steps with regard to research in the area of study or how the study results may be relevant to other populations or contexts. The overall quality of the qualitative studies was in general considered either fair or poor. One of the reasons for a poor judgment was inadequate attention given, in the reporting of the findings of the study, to the acknowledgement of the potential for bias, and limitations of generalizability (Fig. 4).

Studies with a quantitative design were assessed for internal validity and external validity. Studies with a qualitative design were assessed for overall quality and value of the research. Mixed methods studies were assessed for all domains.

## Discussion

Research conducted on MA in high-income countries has been dominated by the US and the UK. The specific regulatory, political and social context of these two countries may have implications for the generalizability of the findings of studies conducted there to other high-income countries.

Another key feature of this literature review is the abundance of clinical research. Although investments in this area of study have advanced understanding of optimal regimens for MA—including the ideal drugs, dose, timing and route of administration—the large quantity of clinical research raises questions about saturation of information in this area. If the findings have converged on a common understanding of optimal regimens, it is possible that additional clinical primary research is unnecessary to further advance this aspect of MA. Indeed, the 2018 publication of the WHO guideline *Medical management of abortion* synetheizes clinical evidence on MA and provides recommendations for timing, dosage, and routes of administration for medications to manage abortion [16]. However, where fundamental gaps in key areas of clinical research persist, investment in clinical studies to should be prioritized. Additional evidence syntheses may be necessary to gain clarity on the specific disparities in MA clinical research.

Future research on the administration of MA medications and management of the MA process should focus on well-defined, under-researched areas, such as innovative models of service delivery that would increase access to women in high-income countries with well-resourced health systems, including online provision, telemedicine, pharmacy provision and home use.

Over 100 of the included studies focused on the assessment of completion of the MA procedure and need for further clinic-based follow-up; and most of these were studies on failure and adverse events. The volume of the literature found on this theme may support investment in systematic reviews to address more specific questions related to completion of the MA procedure.

This review aligns with Kapp et al. call for further research on preferences for MA, particularly regarding more flexible models of service delivery, including pharmacy provision, and home use [17, 18]. Although this literature review identified over 50 studies on women’s preferences and experiences, it is important to understand how women’s preferences vary across population sub-groups to tailor service delivery options to women’s needs and provide a variety of options for how women may access MA. Additional analysis and new studies might be necessary to further develop this area of MA research. The cost-effectiveness of different service delivery models also remains an under-researched area.

The purpose of this review was not only to understand the focus of research in the past decade, but also to gauge the quality of the evidence. The quality assessments suggest that the generalizability of published quantitative research in MA has limitations, since the sample sizes and research settings often restricted the potential applicability of the findings to other contexts. Large-sample, multi-site studies are costly and logistically challenging. However, the research community should question the utility of evidence that holds limited significance beyond a narrow context and consider whether generalizability and broader relevance should become requisite features of future research. Small-scale studies may be important for more in-depth investigation with underrepresented populations, but if so, researchers should be more explicit about the settings and populations under study and whether it is appropriate to generalize the findings to different contexts. If the aim of the research is to generalize broadly, researchers should focus on multi-site research using sampling strategies that increase the representativeness of study populations.

The principles of CerQual informed the markers of quality in qualitative research for this review [15]. Although, it is acknowledged that CerQual is not designed to assess the quality of individual studies, it is also possible that the reporting requirements for publishing the results of qualitative studies in peer-reviewed health science journals are more lenient than criteria outlined in CerQual, and that key features of rigorous qualitative research are simply underreported. Nevertheless, the consequence is a potentially biased body of evidence, with limited wider relevance. Standards for the implementation and publication of qualitative research should be improved within the health sciences, as the potential of such data to drive deeper understanding of complex issues is substantial. Several areas of quantitative research have become more robust and more transparent through the application of quality standards, such as the use of the CONSORT statement for clinical trials, and it is possible that CerQual’s standards will support similar improvements for qualitative research [19].

The strength of this review is its inclusion and assessment of a wide range of literature on MA published in the past decade. However, as the review focused on mapping the characteristics of studies, it could not assess the strength or variability of specific study findings. Furthermore, the gaps in literature were only identified in contrast to the existence of published literature in other contexts. Finally, the studies considered for this review were only assessed for categorization and quality assessment by one researcher. This represents a major limitation. Despite efforts to minimize bias, relying on only one assessment to determine and evaluate the quality of studies is a significant shortcoming of this review. The findings of this literature review should be considered bearing this limitation in mind (Additional file 1).

### Conclusion

Nonetheless, this mapping has identified key gaps in MA research: studies of MA in high-income contexts beyond the US and the UK; models of service delivery, including online provision, telemedicine, pharmacy provision and home use; systematic reviews of literature on completion of the MA procedure; studies on women’s service delivery preferences across various population sub-groups; and cost-effectiveness of different service delivery models. The findings of this review and the identification of these key gaps in the literature can inform an agenda for high-quality, innovative, and rigorous research that would bring the reality of MA in line with long-held aspirations for widespread access.

## Supplementary Information

**Additional file 1.** Included studies reference list.

## Data Availability

The datasets used and/or analysed during the current study are available from the corresponding author on reasonable request.

## Abbreviations

CerQual: Confidence in the Evidence for Reviews of Qualitative research
MA: Medication abortion
RCT: Randomized Controlled Trial
US: United States of America
WHO: World Health Organization
