# Supplementary material for: Glancing at the past and course-setting for the future: lessons from the last decade of research on medication abortion in high-income countries
Source: Reprod Health. 2021 Feb 8;18:30. doi: 10.1186/s12978-021-01081-3 (PMC7869235; doi:10.1186/s12978-021-01081-3)
Supplement: Supplementary file 1 — Additional file 1. Included studies reference list. [file 12978_2021_1081_MOESM1_ESM.docx]

**Included Studies Reference List**

1. Afable-Munsuz A, Gould H, Stewart F, Phillips KA, Van Bebber SL, Moore C. Provider practice models for and costs of delivering medication abortion -- evidence from 11 US abortion care settings. Contraception. 2007;75(1):45–51.
2. Aiken ARA, Digol I, Trussell J, Gomperts R. Self-reported outcomes and adverse events after medical abortion through online telemedicine: population-based study in the Republic of Ireland and Northern Ireland. BMJ 2017; 357:j2011.
3. Aiken ARA, Guthrie KA, Schellekens M, Trussell J, Gomperts R. Barriers to accessing abortion services and perspectives on using mifepristone and misoprostol at home in Great Britain. Contraception 2018; 97(2):177-83.
4. Alsibiani SA. Use of Misoprostol for Self-Induced Medical Abortions among Saudi Women: A Call for Attention. Gynecol Obstet Invest 2014; 78(2):88-93.
5. Amer-Alshiek J, Shiekh O, Agmon A, Grisaru D. What is the right timing for ultrasound evaluation after pregnancy termination with mifepristone? Eur J Obstet Gynecol Reprod Biol. 2015; 189:24-6.
6. Andersson IM, Benson L, Christensson K, Gemzell-Danielsson K. Paracervical block as pain treatment during second-trimester medical termination of pregnancy: an RCT with bupivacaine versus sodium chloride. Hum Reprod 2016; 31(1):67-74.
7. Andersson IM, Gemzell-Danielsson K, Christensson K. Caring for women undergoing second-trimester medical termination of pregnancy. Contraception 2014; 89(5):460-5.
8. Astle H, Cameron ST, Johnstone A. Comparison of unscheduled re-attendance and contraception at discharge, among women having the final stage of early medical abortion at home and those remaining in hospital. J Fam Plann Reprod Health Care 2012; 38(1):35-40.
9. Au HK, Liu CF, Tzeng CR, Chien LW. Association between ultrasonographic parameters of Cesarean scar defect and outcome of early termination of pregnancy. Ultrasound Obstet Gynecol 2016; 47(4):506-10.
10. Avraham S, Gat I, Duvdevani NR, Haas J, Frenkel Y, Seidman DS. Pre-emptive effect of ibuprofen versus placebo on pain relief and success rates of medical abortion: a double-blind, randomized, controlled study. Fertil Steril 2012; 97(3):612-5.
11. Barros Pereira I, Carvalho RM, Graca LM. Intra-abortion contraception with etonogestrel subdermal implant. Eur J Obstet Gynecol Reprod Biol 2015; 185:33-5.
12. Bennett IM, Baylson M, Kalkstein K, Gillespie G, Bellamy SL, Fleischman J. Early abortion in family medicine: clinical outcomes [published correction appears in Ann Fam Med. 2010 Jan-Feb;8(1):84]. Ann Fam Med. 2009;7(6):527–533.
13. Bernard N, Elefant E, Carlier P, Tebacher M, Barjhoux CE, Bos-Thompson MA, et al. Continuation of pregnancy after first-trimester exposure to mifepristone: an observational prospective study. BJOG 2013; 120(5):568-74.
14. Betstadt SJ, Turok DK, Kapp N, Feng KT, Borgatta L. Intrauterine device insertion after medical abortion. Contraception. 2011;83(6):517–521.
15. Bizjak I, Fiala C, Berggren L, Hognert H, Sääv I, Bring J, et al. Efficacy and safety of very early medical termination of pregnancy: a cohort study. BJOG 2017; 124(13):1993-9.
16. Blum J, Shochet T, Lynd K, et al. Can at-home semi-quantitative pregnancy tests serve as a replacement for clinical follow-up of medical abortion? A US study. Contraception. 2012;86(6):757–762.
17. Boersma AA, Meyboom-de Jong B, Kleiverda G. Mifepristone followed by home administration of buccal misoprostol for medical abortion up to 70 days of amenorrhoea in a general practice in Curaçao. Eur J Contracept Reprod Health Care 2011; 16(2):61-6.
18. Bracken H, Lohr PA, Taylor J, Morroni C, Winikoff B. RU OK? The acceptability and feasibility of remote technologies for follow-up after early medical abortion, Contraception 2014; 90(1):29-35.
19. Bracken H, Clark W, Lichtenberg ES, et al. Alternatives to routine ultrasound for eligibility assessment prior to early termination of pregnancy with mifepristone-misoprostol. BJOG. 2011;118(1):17–23.
20. Brouns JF, van Wely M, Burger MP, van Wijngaarden WJ. Comparison of two dose regimens of misoprostol for second-trimester pregnancy termination. Contraception 2010; 82(3):266-75.
21. Cameron S, Glasier A, Dewart H, Johnstone A. Women’s experiences of the final stage of early medical abortion at home: results of a pilot survey. J Fam Plann Reprod Health Care 2010; 36(4):213-6.
22. Cameron ST, Berugoda N, Johnstone A, Glasier A. Assessment of a ‘fast-track’ referral service for intrauterine contraception following early medical abortion. J Fam Plann Reprod Health Care 2012; 38(3):175-8.
23. Cameron ST, Glasier A, Dewart H, Johnstone A, Burnside A. Telephone follow-up and self-performed urine pregnancy testing after early medical abortion: a service evaluation. Contraception 2012; 86(1):67-73.
24. Cameron ST, Glasier A, Johnstone A. Comparison of uptake of long-acting reversible contraception after abortion from a hospital or a community sexual and reproductive healthcare setting: an observational study. J Fam Plann Reprod Health Care 2017; 43(1):31-6.
25. Cameron ST, Glasier A, Johnstone A. Shifting abortion care from a hospital to a community sexual and reproductive health care setting. J Fam Plann Reprod Health Care 2016; 42(2):127-32.
26. Cameron ST, Glasier A, Johnstone A, Dewart H, Campbell A. Can women determine the success of early medical termination of pregnancy themselves? Contraception 2015; 91(1):6-11.
27. Cappiello J, Merrell J, Rentschler D. Women's experience of decision-making with medication abortion. MCN Am J Matern Child Nurs. 2014;39(5):325–330.
28. Carlsson T, Axelsson O. Patient Information Websites About Medically Induced Second-Trimester Abortions: A Descriptive Study of Quality, Suitability, and Issues. J Med Internet Res 2017; 19(1):e8.
29. Carr Ellis S, Kapp N, Vragpvoc O, Borgata L. Randomized trial of buccal versus vaginal misoprostol for induction of second trimester abortion. Contraception. 2010;81(5):441–445.
30. Cavet S, Fiala C, Scemama A, Partouche H. Assessment of pain during medical abortion with home use of misoprostol. Eur J Contracept Reprod Health Care 2017; 22(3):207-11.
31. Chai J, Ho PC. A pilot study on the combined use of letrozole, mifepristone, and misoprostol in termination of first trimester pregnancy up to 9 weeks' gestation. Eur J Obstet Gynecol Reprod Biol 2013; 171(2):291-4.
32. Chai J, Wong CY, Ho PC. A randomized clinical trial comparing the short-term side effects of sublingual and buccal routes of misoprostol administration for medical abortions up to 63 days’ gestation. Contraception 2013; 87(4):480-5.
33. Chen MJ, Rounds KM, Creinin MD, Cansino C, Hou MY. Comparing office and telephone follow-up after medical abortion. Contraception. 2016;94(2):122–126.
34. Cheng SY, Hsue CS, Hwang GH, Tsai LC, Pei SC. Hourly oral misoprostol administration for terminating midtrimester pregnancies: a pilot study. Taiwan J Obstet Gynecol 2010; 49(4):438-41.
35. Chien LW, Liu WM, Tzeng CR, Au HK. Effect of Previous Live Birth and Prior Route of Delivery on the Outcome of Early Medical Abortion. Obstet Gynecol 2009; 113(3):669-74.
36. Chong E, Frye LJ, Castle J, Dean G, Kuehl L, Winikoff B. A prospective, non-randomized study of home use of mifepristone for medical abortion in the U.S. Contraception. 2015;92(3):215–219.
37. Chou SY, Chen CY, Hsu MI, Chow PK, Hsu CS, Chiang HK. Sonographic quantification of endometrial changes after abortion with computer-assisted image analysis. Acta Obstet Gynecol Scand 2010; 89(3):385-9.
38. Church E, Sengupta S, Chia KV. The contraceptive implant for long acting reversible contraception in patients undergoing first trimester medical termination of pregnancy. Sex Reprod Healthc 2010; 1(3):105-9.
39. Clark W, Bracken H, Tanenhaus J, Schweikert S, Lichtenberg ES, Winikoff B. Alternatives to a routine follow-up visit for early medical abortion. Obstet Gynecol. 2010;115(2 Pt 1):264–272.
40. Clark W, Panton T, Hann L, Gold M. Medication abortion employing routine sequential measurements of serum hCG and sonography only when indicated. Contraception. 2007;75(2):131–135.
41. Cleland K, Creinin MD, Nucatola D, Nshom M, Trussell J. Significant adverse events and outcomes after medical abortion. Obstet Gynecol. 2013;121(1):166–171.
42. Coles MS, Makino KK, Phelps R. Knowledge of medication abortion among adolescent medicine providers. J Adolesc Health. 2012;50(4):383–388.
43. Creinin MD, Schreiber CA, Bednarek P, et al. Mifepristone and misoprostol administered simultaneously versus 24 hours apart for abortion: a randomized controlled trial. Obstet Gynecol. 2007;109(4):885–894.
44. Dawson AJ, Nicolls R, Bateson D, Doab A, Estoesta J, Brassil A, et al. Medical termination of pregnancy in general practice in Australia: a descriptive-interpretive qualitative study. Reprod Health 2017; 14(1):39.
45. Dayananda I, Maurer R, Fortin J, Goldberg AB. Medical abortion follow-up with serum human chorionic gonadotropin compared with ultrasonography: a randomized controlled trial. Obstet Gynecol. 2013;121(3):607–613.
46. De Costa C, Douglas H, Black K. Making it legal: Abortion providers’ knowledge and use of abortion law in New South Wales and Queensland. Aust N Z J Obstet Gynaecol 2013; 53(2):184-9.
47. Dehlendorf CE, Fox EE, Ali RF, Anderson NC, Reed RD, Lichtenberg ES. Medication abortion failure in women with and without previous cesarean delivery. Contraception. 2015;92(5):463–468.
48. Di Carlo C, Savoia F, Ferrara C, Sglavo G, Tommaselli GA, Giampaolino P, et al. "In patient" medical abortion versus surgical abortion: patient's satisfaction. Gynecol Endocrinol 2016; 32(8):650-4.
49. Di Carlo C, Savoia F, Morra I, Ferrara C, Sglavo G, Nappi C. Effects of a prolonged, 72 hours, interval between mifepristone and gemeprost in second trimester termination of pregnancy: a retrospective analysis. Gynecol Endocrinol 2014; 30(8):605-7.
50. Downing S, McNamee H, Penney D, Leamy J, de Costa C, Russell DB. Three years on: a review of medical terminations of pregnancy performed in a sexual health service. Sex Health 2010; 7(2):212-5.
51. Dunn S, Panjwani D, Gupta M, Meaney C, Morgan R, Feuerstein E. Comparison of remote and in-clinic follow-up after methotrexate/misoprostol abortion. Contraception 2015; 92(3):220-6.
52. Fiol V, Briozzo L, Labandera A, Recchi V, Piñeyro M. Improving care of women at risk of unsafe abortion: Implementing a risk-reduction model at the Uruguayan-Brazilian border. Int J Gynaecol Obstet 2012; 118 Suppl 1:S21-7.
53. Fiol V, Rieppi L, Aguirre R, Nozar M, Gorgoroso M, Coppola F, et al. The role of medical abortion in the implementation of the law on voluntary termination of pregnancy in Uruguay. Int J Gynaecol Obstet 2016; 134(S1):S12-5.
54. Finer LB, Wei J. Effect of mifepristone on abortion access in the United States. Obstet Gynecol. 2009;114(3):623–630.
55. Fjerstad M, Sivin I, Lichtenberg ES, Trussell J, Cleland K, Cullins V. Effectiveness of medical abortion with mifepristone and buccal misoprostol through 59 gestational days. Contraception. 2009;80(3):282–286.
56. Fjerstad M, Trussell J, Lichtenberg ES, Sivin I, Cullins V. Severity of infection following the introduction of new infection control measures for medical abortion. Contraception. 2011;83(4):330–335.
57. Fjerstad M, Trussell J, Sivin I, Lichtenberg ES, Cullins V. Rates of serious infection after changes in regimens for medical abortion. N Engl J Med. 2009;361(2):145–151.
58. Freeman MD, Porat N, Rojansky N, Elami-Suzin M, Winograd O, Ben-Meir A. Physical symptoms and emotional responses among women undergoing induced abortion protocols during the second trimester. Int J Gynaecol Obstet 2016; 135(2):154-7.
59. Frye LJ, Chong E, Winikoff B; NCT01799252 Trial Investigators. What happens when we routinely give doxycycline to medical abortion patients?. Contraception. 2015;91(1):19–24.
60. Fuchs N, Maymon R, Ben-Ami I, Mendlovic S, Schneider D, Pansky M, et al. Clinical, surgical, and histopathologic outcomes following failed medical abortion. Int J Gynaecol Obstet 2012; 117(3):234-8.
61. Gatter M, Cleland K, Nucatola DL. Efficacy and safety of medical abortion using mifepristone and buccal misoprostol through 63 days. Contraception. 2015;91(4):269–273.
62. Gaudu S, Crost M, Esterle L. Results of a 4-year study on 15,447 medical abortions provided by privately practicing general practitioners and gynecologists in France. Contraception 2013; 87(1):45-50.
63. Godfrey EM, Bordoloi A, Moorthie M, Pela E. Medication abortion within a student health care clinic: a review of the first 46 consecutive cases. J Am Coll Health. 2012;60(2):178–183.
64. Godfrey EM, Anderson A, Fielding SL, Meyn L, Creinin MD. Clinical utility of urine pregnancy assays to determine medical abortion outcome is limited. Contraception. 2007;75(5):378–382.
65. Goldstone P, Michelson J, Williamson E. Effectiveness of early medical abortion using low-dose mifepristone and buccal misoprostol in women with no defined intrauterine gestational sac. Contraception 2013; 87(6):855-8.
66. Goldstone P, Michelson J, Williamson E. Early medical abortion using low-dose mifepristone followed by buccal misoprostol: a large Australian observational study. Med J Aust 2012 3; 197(5):282-6.
67. Goldstone P, Walker C, Hawtin K. Efficacy and safety of mifepristone–buccal misoprostol for early medical abortion in an Australian clinical setting. Aust N Z J Obstet Gynaecol 2017; 57(3):366-71.
68. Gomez O, Borrás A, Rabanal A, Palacio M, Carceller A, Coll O, Gratacós E. Mifepristone–misoprostol midtrimester abortion: impact of gestational age on the induction-to-abortion interval. Contraception 2010; 81(2):97-101.
69. Gomperts R, Petow SA, Jelinska K, Steen L, Gemzell-Danielsson K, Kleiverda G. Regional differences in surgical intervention following medical termination of pregnancy provided by telemedicine. Acta Obstet Gynecol Scand 2012; 91(2):226-31.
70. Gomperts RJ, Jelinska K, Davies S, Gemzell-Danielsson K, Kleiverda G. Using telemedicine for termination of pregnancy with mifepristone and misoprostol in settings where there is no access to safe services. BJOG 2008; 115(9):1171-5.
71. Goodyear-Smith F, Knowles A. Choosing medical or surgical terminations of pregnancy in the first trimester: What is the difference? Aust N Z J Obstet Gynaecol 2009; 49(2):211-5.
72. Grapsas X, Liberis V, Vassaras G, Tsikouras P, Vlachos G, Galazios G. Misoprostol and first trimester pregnancy termination. Clin Exp Obstet Gynecol 2008; 35(1):32-4.
73. Grindlay K, Grossman D. Telemedicine provision of medical abortion in Alaska: Through the provider's lens. J Telemed Telecare. 2017;23(7):680–685.
74. Grindlay K, Lane K, Grossman D. Women's and providers' experiences with medical abortion provided through telemedicine: a qualitative study. Women’s Health Issues. 2013;23(2):e117–e122.
75. Grossman DA, Grindlay K, Buchacker T, Potter JE, Schmertmann CP. Changes in service delivery patterns after introduction of telemedicine provision of medical abortion in Iowa. Am J Public Health. 2013;103(1):73–78.
76. Grossman D, Grindlay K. Safety of Medical Abortion Provided Through Telemedicine Compared With In Person. Obstet Gynecol. 2017;130(4):778–782.
77. Grossman D, Grindlay K, Buchacker T, Lane K, Blanchard K. Effectiveness and acceptability of medical abortion provided through telemedicine. Obstet Gynecol. 2011;118(2 Pt 1):296–303.
78. Gupta S, Kapwepwe S. Collaboration with the voluntary sector in setting up an early medical abortion service in the PCT. J Obstet Gynaecol 2007; 27(5):506-9.
79. Haimov-Kochman R, Arbel R, Sciaky-Tamir Y, Brzezinski A, Laufer N, Yagel S. Risk factors for unsuccessful medical abortion with mifepristone and misoprostol. Acta Obstet Gynecol Scand 2007; 86(4):462-6.
80. Hassoun D, Perin I, Hien H, Demars HH. Feasibility of self-performed urine pregnancy testing for follow-up after medical abortion. Eur J Obstet Gynecol Reprod Biol 2016; 197:174-8.
81. Hayes JL, Achilles SL, Creinin MD, Reeves MF. Outcomes of medical abortion through 63 days in women with twin gestations. Contraception. 2011;84(5):505–507.
82. Hedqvist M, Brolin L, Tyden T, Larsson M. Women’s experiences of having an early medical abortion at home. Sex Reprod Healthc 2016; 9:48-54.
83. Heikinheimo O, Leminen R, Suhonen S. Termination of early pregnancy using flexible, low-dose mifepristone–misoprostol regimens. Contraception 2007; 76(6):456-60.
84. Hognert H, Kopp Kallner H, Cameron S, Nyrelli C, Jawad I, Heller R, et al. Immediate versus delayed insertion of an etonogestrel releasing implant at medical abortion—a randomized controlled equivalence trial. Hum Reprod 2016;31(11):2484-90.
85. Horning EL, Chen BA, Meyn LA, Creinin MD. Comparison of medical abortion follow-up with serum human chorionic gonadotropin testing and in-office assessment. Contraception. 2012;85(4):402–407.
86. Joensuu-Manninen H, Kuvaja P, Talvensaari-Mattila A. Clinical efficacy of mifepristone and misoprostol in second trimester pregnancy termination. Acta Obstet Gynecol Scand 2010; 89(12):1552-6.
87. Jones HE, O’Connell White K, Norman WV, Guilbert E, Lichtenberg ES, Paul M. First trimester medication abortion practice in the United States and Canada. PLoS ONE 2016; 12(10):e0186487.
88. Ireland LD, Gatter M, Chen AY. Medical Compared With Surgical Abortion for Effective Pregnancy Termination in the First Trimester. Obstet Gynecol. 2015;126(1):22–28.
89. Jackson AV, Dayananda I, Fortin JM, Fitzmaurice G, Goldberg AB. Can women accurately assess the outcome of medical abortion based on symptoms alone?. Contraception. 2012;85(2):192–197.
90. Kapp N, Borgatta L, Stubblefield P, Vragovic O, Moreno N. Mifepristone in second-trimester medical abortion: a randomized controlled trial. Obstet Gynecol. 2007;110(6):1304–1310.
91. Kelly T, Suddes J, Howel D, Hewison J, Robson S. Comparing medical versus surgical termination of pregnancy at 13–20 weeks of gestation: a randomised controlled trial. BJOG 2010; 117(12):1512-20.
92. Kero A, Wulff M, Lalos A. Home abortion implies radical changes for women. Eur J Contracept Reprod Health Care 2009; 14(5):324-33.
93. Koh DSC, Ang EPJ, Coyuco JC, Teo HZ, Huang X, Wei X, et al. Comparing two regimens of intravaginal misoprostol with intravaginal gemeprost for second-trimester pregnancy termination: a randomised controlled trial. J Fam Plann Reprod Health Care 2017; 43(4):252-9.
94. Kopp Kallner H, Gomperts R, Salmonsson E, Johansson M, Marions L, Gemzell-Danielsson K. The efficacy, safety and acceptability of medical termination of pregnancy provided by standard care by doctors or by nurse-midwives: a randomised controlled equivalence trial. BJOG 2015; 122(4):510-7.
95. Kopp Kallner H, Fiala C, Stephansson O, Gemzell-Danielsson K. Home self-administration of vaginal misoprostol for medical abortion at 50–63 days compared with gestation of below 50 days. Hum Reprod 2010; 25(5):1153-7.
96. Kopp Kallner H, Fiala C, Gemzell-Danielsson K. Assessment of significant factors affecting acceptability of home administration of misoprostol for medical abortion. Contraception. 2012; 85(4):394-7.
97. Korjamo R, Mentula M, Heikinheimo O. Fast-track vs. delayed insertion of the levonorgestrel-releasing intrauterine system after early medical abortion — a randomized trial. Contraception 2017; 96(5):344-351.
98. Korjamo R, Mentula M, Heikinheimo O. Immediate versus delayed initiation of the levonorgestrel-releasing intrauterine system following medical termination of pregnancy – 1 year continuation rates: a randomised controlled trial. BJOG 2017; 124(13):1957-64.
99. Laursen L, Stumbras K, Lewnard I, Haider S. Contraceptive Provision after Medication and Surgical Abortion. Womens Health Issues. 2017;27(5):546–550.
100. Lee VC, Tang OS, Ng EH, Yeung WS, Ho PC. A pilot study on the use of letrozole with either misoprostol or mifepristone for termination of pregnancy up to 63 days. Contraception 2011; 83(1):62-7.
101. Lee VC, Tang OS, Ng EH, Yeung WS, Ho PC. A prospective double-blinded, randomized, placebo-controlled trial on the use of letrozole pretreatment with misoprostol for second-trimester medical abortion. Contraception 2011; 84(6):628-33.
102. Lee RY, Moles R, Chaar B. Mifepristone (RU486) in Australian pharmacies: the ethical and practical challenges. Contraception 2015; 91(1):25-30.
103. Leeman L, Asaria S, Espey E, Ogburn J, Gopman S, Barnett S. Can mifepristone medication abortion be successfully integrated into medical practices that do not offer surgical abortion?. Contraception. 2007;76(2):96–100.
104. Lefebvre P, Cotte M, Monniez N, Norel G. The role of parity in medical abortion up to 49 days of amenorrhoea. Eur J Contracept Reprod Health Care 2008; 13(4):404-11.
105. Leone Roberti Maggiore U, Silanos R, Carlevaro S, Gratarola A, Venturini PL, Ferrero S, et al. Programmed intermittent epidural bolus versus continuous epidural infusion for pain relief during termination of pregnancy: a prospective, double-blind, randomized trial. Int J Obstet Anesth 2016; 25:37-44.
106. Les K, Gomperts R, Gemzell-Danielsson K. Experiences of women living in Hungary seeking a medical abortion online. Eur J Contracept Reprod Health Care 2017; 22(5):360-2.
107. Li YT, Hou GQ, Chen TH, Chu YC, Lin TC, Kuan LC, et al. High-dose misoprostol as an alternative therapy after failed medical abortion. Taiwan J Obstet Gynecol 2008; 47(4):408-11.
108. Li YT, Hsieh JC, Hou GQ, Chen TH, Chu YC, Lin TC, et al. Simultaneous use of mifepristone and misoprostol for early pregnancy termination. Taiwan J Obstet Gynecol 2011; 50(1):11-4.
109. Livshits A, Machtinger R, David LB, Spira M, Moshe-Zahav A, Seidman DS. Ibuprofen and paracetamol for pain relief during medical abortion: a double-blind randomized controlled study. Fertil Steril 2009; 91(5):1877-80.
110. Lo SS, Ho PC. First-trimester medical abortion service in Hong Kong. Hong Kong Med J 2015; 21(5):462-7.
111. Lohr PA, Reeves MF, Hayes JL, Harwood B, Creinin MD. Oral mifepristone and buccal misoprostol administered simultaneously for abortion: a pilot study. Contraception. 2007;76(3):215–220.
112. Lohr PA, Wade J, Riley L, Fitzgibbon A, Furedi A. Women's opinions of the home management of early medical abortion in the UK. J Fam Plann Reprod Health Care 2010; 36(1):21-5.
113. Løkeland M, Bjørge T, Iversen OE, Akerkar R, Bjørge L. Implementing medical abortion with mifepristone and misoprostol in Norway 1998–2013. Int J Epidemiol 2017; 46(2):643-651.
114. Løkeland M, Iversen OE, Engeland A, Økland I, Bjørge L. Medical abortion with mifepristone and home administration of misoprostol up to 63 days’ gestation. Acta Obstet Gynecol Scand 2014; 93(7):647-53.
115. Løkeland M, Iversen OE, Dahle GS, Nappen MH, Ertzeid L, Bjørge L. Medical Abortion at 63 to 90 Days of Gestation. Obstet Gynecol 2010; 115(5):962-8.
116. Mählck CG, Bäckström T. Follow-up after early medical abortion: Comparing clinical assessment with self-assessment in a rural hospital in northern Norway. Eur J Obstet Gynecol Reprod Biol 2017; 213:1-3.
117. Makenzius M, Tydén T, Darj E, Larsson M. Autonomy and dependence – experiences of home abortion, contraception and prevention. Scand J Caring Sci 2013; 27(3):569-79.
118. Männistö J, Mentula M, Bloigu A, Gissler M, Niinimäki M, Heikinheimo O. Medical termination of pregnancy during the second versus the first trimester and its effects on subsequent pregnancy. Contraception 2014; 89(2):109-15.
119. Matia MG, Trumper EC, Fures NO, Orchuela J. A replication of the Uruguayan model in the province of Buenos Aires, Argentina, as a public policy for reducing abortion-related maternal mortality. Int J Gynaecol Obstet 2016; 134 Suppl 1:S31-4.
120. McKay RJ, Rutherford L. Women ’s satisfaction with early home medical abortion with telephone follow-up: A questionnaire-based study in the UK. J Obstet Gynaecol 2013; 33(6):601-4.
121. Mentula M, Heikinheimo O. Risk factors of sugical evacuation following second-trimester medical termination of pregnancy. Contraception 2012; 86(2):141-6.
122. Mentula M, Suhonen S, Heikinheimo O. One- and two-day dosing intervals between mifepristone and misoprostol in second trimenter medical termination of pregnancy-a randomized trial. Hum Reprod 2011; 26(10):2690-7.
123. Mentula MJ, Niinimäki M, Suhonen S, Hemminki E, Gissler M, Heikinheimo O. Immediate adverse events after second trimester medical termination of pregnancy: results of a nationwide registry study. Hum Reprod 2011; 26(4):927-32.
124. Michie L, Cameron ST. Simplified follow-up after early medical abortion: 12-month experience of a telephone call and self-performed low-sensitivity urine pregnancy test. Contraception 2014; 89(5):440-5.
125. Michie L, Cameron ST, Glasier A. Abortion care services delivered from a community sexual and reproductive health setting: views of health care professionals. J Fam Plann Reprod Health Care 2013; 39(4):270-5.
126. Mirmilstein V, Rowlands S, King JF. Outcomes for subsequent pregnancy in women who have undergone misoprostol mid-trimester termination of pregnancy. Aust N Z J Obstet Gynaecol 2009; 49(2):195-7.
127. Mizuno M. Clinical experience and perception of abortion: A cross-sectional survey of gynecologists in Japan. Sex Reprod Healthc 2015; 6(4):255-6.
128. Moreau C, Trussell J, Desfreres J, Bajos N. Medical vs. surgical abortion: the importance of women’s choice. Contraception 2011; 84(3):224-9.
129. Morotti M, Calanni L, Gianola G, Anserini P, Venturini PL, Ferrero S. Changes in Sexual Function after Medical or Surgical Termination of Pregnancy. J Sex Med 2014; 11(6):1495-504.
130. Mulligan E, Messenger H. Mifepristone in South Australia: the first 1343 tablets. Aust Fam Physician 2011; 40(5):342-5.
131. Murtagh C, Wells E, Raymond EG, Coeytaux F, Winikoff B. Exploring the feasibility of obtaining mifepristone and misoprostol from the internet. Contraception. 2018;97(4):287–291.
132. Myers AJ, Lohr PA, Pfeffer N. Disposal of fetal tissue following elective abortion: what women think. J Fam Plann Reprod Health Care 2015; 41(2):84-9.
133. Myran DT, Carew CL, Tang J, Whyte H, Fisher WA. Medical Students’ Intentions to Seek Abortion Training and to Provide Abortion Services in Future Practice. J Obstet Gynaecol Can 2015; 37(3):236-44.
134. Napolitano R, Ghosh M, Gillott DJ, Ojha K. Three-Dimensional Doppler Sonography in Asymptomatic and Symptomatic Women After Medical Termination of Pregnancy. J Ultrasound Med 2014; 33(5):847-52.
135. Newton D, Bayly C, McNamee K, Bismark M, Hardiman A, Webster A, et al. "…a one stop shop in their own community": Medical abortion and the role of general practice. Aust N Z J Obstet Gynaecol 2016; 56(6):648-54.
136. Newton D, Bayly C, McNamee K, Hardiman A, Bismark M, Webster A, et al. How do women seeking abortion choose between surgical and medical abortion? Perspectives from abortion service providers. Aust N Z J Obstet Gynaecol 2016; 56(5):523-9.
137. Niinimäki M, Pouta A, Bloigu A, Gissler M, Hemminki E, Suhonen S, et al. Immediate Complications After Medical Compared With Surgical Termination of Pregnancy. Obstet Gynecol 2009; 114(4):795-804.
138. Niinimäki M, Pouta A, Bloigu A, Gissler M, Hemminki E, Suhonen S, et al. Frequency and Risk Factors for Repeat Abortions After Surgical Compared With Medical Termination of Pregnancy. Obstet Gynecol 2009; 113(4):845-52.
139. Niinimäki M, Suhonen S, Mentula M, Hemminki E, Heikinheimo O, Gissler M. Comparison of rates of adverse events in adolescent and adult women undergoing medical abortion: population register based study. BMJ 2011; 342:d2111.
140. Nijman TA, Voogdt KG, Teunissen PW, van der Voorn PJ, de Groot CJ, Bakker PC. Association between infection and fever in terminations of pregnancy using misoprostol: a retrospective cohort study. BMC Pregnancy Childbirth 2017; 17(1):7.
141. Nilas L, Glavind-Kristensen M, Vejborg T, Knudsen UB. One or two day mifepristone-misoprostol interval for a second trimester abortion. Acta Obstet Gynecol Scand 2007; 86(9):1117-21.
142. Nisand I, Bettahar K; Investigators of the aMaYa Study. Medical management of unwanted pregnancy in France: modalities and outcomes. The aMaYa study. Eur J Obstet Gynecol Reprod Biol 2015; 184:13-8.
143. Obata-Yasuoka M, Hamada H, Watanabe H, Shimura R, Toyoda M, Yagi H, et al. Midtrimester termination of pregnancy using gemeprost in combination with laminaria in women who have previously undergone cesarean section. J Obstet Gynaecol Res 2009; 35(5):901-5.
144. Odeh M, Tendler R, Sosnovsky V, Kais M, Ophir E, Bornstein J. The Effect of Parity and Gravidity on the Outcome of Medical Termination of Pregnancy. Isr Med Assoc J 2010; 12(10):606-8.
145. Ojha K, Gillott DJ, Wood P, Valcarcel E, Matah A, Talaulikar VS. Clinical outcomes from a prospective study evaluating the role of ambulation during medical termination of pregnancy. Contraception 2012; 85(4):398-401.
146. Oppegaard KS, Qvigstad E, Fiala C, Heikinheimo O, Benson L, Gemzell-Danielsson K. Clinical follow-up compared with self-assessment of outcome after medical abortion: a multicentre, non-inferiority, randomised, controlled trial. Lancet 2015; 385(9969):698-704.
147. Page C, Stumbar S, Gold M. Attitudes and preferences toward the provision of medication abortion in an urban academic internal medicine practice. J Gen Intern Med. 2012;27(6):647–652.
148. Palma Manriquez I, Moreno Standen C, Álvarez Carimoney A, Richards A. Experience of clandestine use of medical abortion among university students in Chile: a qualitative study. Contraception 2018; 97(2):100-7.
149. Parashar P, Iversen OE, Midbøe G, Myking O, Bjørge L. Medical abortion in the first trimester: The use of serum hCG and endometrial thickness as markers of completeness. Eur J Contracept Reprod Health Care 2007; 12(4):366-71.
150. Pazol K, Creanga AA, Zane SB. Trends in use of medical abortion in the United States: reanalysis of surveillance data from the Centers for Disease Control and Prevention, 2001-2008. Contraception. 2012;86(6):746–751.
151. Perriera LK, Reeves MF, Chen BA, Hohmann HL, Hayes J, Creinin MD. Feasibility of telephone follow-up after medical abortion. Contraception. 2010;81(2):143–149.
152. Perrin E, Berthoud M, Pott M, Vera AG, Bianchi-Demicheli F. Views of healthcare professionals dealing with legal termination of pregnancy up to 12 WA in French-speaking Switzerland. Swiss Med Wkly 2012; 142:w13584.
153. Pheterson G, Azize Y. Abortion within and around the law in the Caribbean. P R Health Sci J 2008; 27(1):93-9.
154. Pocius KD, Maurer R, Fortin J, Goldberg AB, Bartz D. Early serum human chorionic gonadotropin (hCG) trends after medication abortion. Contraception. 2015;91(6):503–506.
155. Pocius KD, Bartz D, Maurer R, Stenquist A, Fortin J, Goldberg AB. Serum human chorionic gonadotropin (hCG) trend within the first few days after medical abortion: a prospective study. Contraception. 2017;95(3):263–268.
156. Pohjoranta E, Suhonen S, Mentula M, Heikinheimo O. Intrauterine contraception after medical abortion: factors affecting success of early insertion. Contraception 2017; 95(3):257-62.
157. Pohjoranta E, Suhonen S, Heikinheimo O. Attendance at post-abortal follow-up visits is low – can the risks of non-attendance be identified? Acta Obstet Gynecol Scand 2011; 90(5):543-6.
158. Prine L, Shannon C, Gillespie G, et al. Medical abortion: outcomes in a family medicine setting. J Am Board Fam Med. 2010;23(4):509–513.
159. Purcell C, Brown A, Melville C, McDaid LM. Women’s embodied experiences of second trimester medical abortion. Fem Psychol 2017; 27(2):163-85.
160. Purcell C, Cameron S, Lawton J, Glasier A, Harden J. Contraceptive care at the time of medical abortion: experiences of women and health professionals in a hospital or community sexual and reproductive health context. Contraception 2016; 93(2):170-7.
161. Purcell C, Cameron S, Lawton J, Glasier A, Harden J. Self-management of first trimester medical termination of pregnancy: a qualitative study of women's experiences. BJOG 2017; 124(13):2001-8.
162. Raymond EG, Weaver MA, Louie KS, et al. Effects of Depot Medroxyprogesterone Acetate Injection Timing on Medical Abortion Efficacy and Repeat Pregnancy: A Randomized Controlled Trial. Obstet Gynecol. 2016;128(4):739–745.
163. Raymond EG, Weaver MA, Louie KS, et al. Prophylactic compared with therapeutic ibuprofen analgesia in first-trimester medical abortion: a randomized controlled trial. Obstet Gynecol. 2013;122(3):558–564.
164. Reeves MF, Fox MC, Lohr PA, Creinin MD. Endometrial thickness following medical abortion is not predictive of subsequent surgical intervention. Ultrasound Obstet Gynecol. 2009;34(1):104–109.
165. Reeves MF, Monmaney JA, Creinin MD. Predictors of uterine evacuation following early medical abortion with mifepristone and misoprostol. Contraception. 2016;93(2):119–125.
166. Robson SC, Kelly T, Howel D, Deverill M, Hewison J, Lie ML, et al. Randomised preference trial of medical versus surgical termination of pregnancy less than 14 weeks’ gestation (TOPS). Health Technol Assess 2009; 13(53):1-124, iii-iv.
167. Rocca CH, Goodman S, Grossman D, et al. Contraception after medication abortion in the United States: results from a cluster randomized trial. Am J Obstet Gynecol. 2018;218(1):107.e1–107.e8.
168. Rooney KA, Denny AE, Hou MY, Creinin MD. LARC utilization based on type of medical abortion follow-up at an academic center. Contraception. 2015;91(5):403–405.
169. Rousset C, Brulfert C, Séjourné N, Goutaudier N, Chabrol H. Posttraumatic Stress Disorder and psychological distress following medical and surgical abortion. J Reprod Infant Psychol 2012; 29(5):506-17.
170. Sääv I, Stephansson O, Gemzell-Danielsson K. Early versus Delayed Insertion of Intrauterine Contraception after Medical Abortion — A Randomized Controlled Trial. PLoS One 2012; 7(11):e48948.
171. Sääv I, Fiala C, Hämäläinen JM, Heikinheimo O, Gemzell-Danielsson K. Medical abortion in lactating women - low levels of mifepristone in breast milk. Acta Obstet Gynecol Scand 2010; 89(5):618-22.
172. Samuel MI, Parsons JH. Hygroscopic dilator (Dilapan-S™) and misoprostol combination for the early first-trimester termination of pregnancy: a pilot study. J Fam Plann Reprod Health Care 2009; 35(1):45-7.
173. Saurel-Cubizolles MJ, Opatowski M, David P, Bardy F, Dunbavand A. Pain during medical abortion: a multicenter study in France. Eur J Obstet Gynecol Reprod Biol 2015; 194:212-7.
174. Schreiber CA, Sober S, Ratcliffe S, Creinin MD. Ovulation resumption after medical abortion with mifepristone and misoprostol. Contraception. 2011;84(3):230–233.
175. Scioscia M, Vimercati A, Pontrelli G, Nappi L, Selvaggi L. Patients’ obstetric history in mid-trimester termination of pregnancy with gemeprost: Does it really matter? Eur J Obstet Gynecol Reprod Biol 2007; 130(1):42-5.
176. Shankar M, Black KI, Goldstone P, Hussainy S, Mazza D, Petersen K, et al. Access, equity and cost of induced abortion services in Australia: a cross-sectional study. Aust N Z J Public Health 2017; 41(3):309-14.
177. Sheinfeld L, Arnott G, El-Haddad J, Foster AM. Assessing abortion coverage in nurse practitioner programs in Canada: a national survey of program directors. Contraception 2016; 94(5):483-8.
178. Sherman S, Harden J, Cattanach D, Cameron ST. Providing experiential information on early medical abortion: a qualitative evaluation of an animated personal account, Lara’s Story. J Fam Plann Reprod Health Care 2017; 43(4):269-273.
179. Shimoni N, Davis A, Ramos ME, Rosario L, Westhoff C. Timing of copper intrauterine device insertion after medical abortion: a randomized controlled trial. Obstet Gynecol. 2011;118(3):623–628.
180. Shochet T, Trussell J. Determinants of demand: method selection and provider preference among US women seeking abortion services. Contraception. 2008;77(6):397–404.
181. Singh M, Porter C, Griffiths S. First trimester medical termination of pregnancy: The Nottingham experience. J Obstet Gynaecol 2008; 28(3):315-6.
182. Situ KC, Hemminki E, Gissler M, Virtanen SM, Klemetti R. Perinatal outcomes after induced termination of pregnancy by methods: A nationwide register-based study of first births in Finland 1996-2013. PLoS One 2017; 12(9):e0184078.
183. Sjöström S, Kopp Kallner H, Simeonova E, Madestam A, Gemzell-Danielsson K. Medical Abortion Provided by Nurse-Midwives or Physicians in a High Resource Setting: A Cost-Effectiveness Analysis. PLoS ONE 2016; 11(6):e0158645.
184. Slunska P, Hanacek J, Fanta M, Sehnal B, Gerychová R, Hola A, et al. Management of Medical Termination of Pregnancy (MToP) up until the 7th week of gestation in the Czech Republic. Ceska Gynekol 2017; 82(5):1-8.
185. Sonalkar S, Hou MY, Borgatta L. Administration of the etonogestrel contraceptive implant on the day of mifepristone for medical abortion: a pilot study [published correction appears in Contraception. 2014 Feb;89(2):142. Hou, Melody [corrected to Hou, Melody Y]]. Contraception. 2013;88(5):671–673.
186. Sonalkar S, McClusky J, Hou MY, Borgatta L. Administration of depot medroxyprogesterone acetate on the day of mifepristone for medical abortion: a pilot study. Contraception. 2015;91(2):174–177.
187. Sonalkar S, Ogden SN, Tran LK, Chen AY. Comparison of complications associated with induction by misoprostol versus dilation and evacuation for second-trimester abortion. Int J Gynaecol Obstet. 2017;138(3):272–275.
188. Strafford MA, Mottl-Santiago J, Savla A, Soodoo N, Borgatta L. Relationship of obesity to outcome of medical abortion. Am J Obstet Gynecol. 2009;200(5):e34–e36.
189. Swica Y, Chong E, Middleton T, et al. Acceptability of home use of mifepristone for medical abortion. Contraception. 2013;88(1):122–127.
190. Teal SB, Harken T, Sheeder J, Westhoff C. Efficacy, acceptability and safety of medication abortion in low-income, urban Latina women. Contraception. 2009;80(5):479–483.
191. Teal SB, Dempsey-Fanning A, Westhoff C. Predictors of acceptability of medication abortion. Contraception. 2007;75(3):224–229.
192. Tendler R, Bornstein J, Kais M, Masri I, Odeh M. Early versus late misoprostol administration after mifeprestone for medical abortion. Arch Gynecol Obstet 2015; 292(5):1051-4.
193. Ting WH, Peng FH, Lin HH, Lu HF, Hsiao SM. Factors influencing the abortion interval of second trimester pregnancy termination using misoprostol. Taiwan J Obstet Gynecol 2015; 54(4):408-11.
194. Trussell J, Nucatola D, Fjerstad M, Lichtenberg ES. Reduction in infection-related mortality since modifications in the regimen of medical abortion. Contraception. 2014;89(3):193–196.
195. Tu YA, Chen CL, Lai YL, Lin SY, Lee CN. Transcervical double-balloon catheter as an alternative and salvage method for medical termination of pregnancy in midtrimester. Taiwan J Obstet Gynecol 2017; 56(1):77-80.
196. Tzeng CR, Hwang JL, Au HK, Chien LW. Sonographic patterns of the endometrium in assessment of medical abortion outcomes. Contraception 2013; 88(1):153-9.
197. Umranikar A, Umranikar S. Efficacy of Single Dose of Mifepristone Combined with Two Doses of Misoprostol in Early Medical Abortions. Contraception 21(1):35-40.
198. Upadhyay UD, Johns NE, Combellick SL, Kohn JE, Keder LM, Roberts SC. Comparison of Outcomes before and after Ohio's Law Mandating Use of the FDA-Approved Protocol for Medication Abortion: A Retrospective Cohort Study. PLoS Med. 2016;13(8):e1002110.
199. Virk J, Zhang J, Olsen J. Medical Abortion and the Risk of Subsequent Adverse Pregnancy Outcomes. N Engl J Med 2007; 357(7):648-53.
200. Vitner D, Deutsch M, Paz Y, Khatib T, Bailtiter T, Rosenberg S, et al. Association between gestational age and induction-to-abortion interval in mid-trimester pregnancy termination using misoprostol. Eur J Obstet Gynecol Reprod Biol 2011; 156(2):140-3.
201. Vogel KI, LaRoche KJ, El-Haddad J, Chaumont A, Foster AM. Exploring Canadian women's knowledge of and interest in mifepristone: results from a national qualitative study with abortion patients. Contraception 2016; 94(2):137-42.
202. Wiegerinck MM, Jones HE, O'Connell K, Lichtenberg ES, Paul M, Westhoff CL. Medical abortion practices: a survey of National Abortion Federation members in the United States. Contraception. 2008;78(6):486–491.
203. Winikoff B, Dzuba IG, Chong E, et al. Extending outpatient medical abortion services through 70 days of gestational age. Obstet Gynecol. 2012;120(5):1070–1076.
204. Winikoff B, Dzuba IG, Creinin MD, et al. Two distinct oral routes of misoprostol in mifepristone medical abortion: a randomized controlled trial. Obstet Gynecol. 2008;112(6):1303–1310.
205. Wallin Lundell I, Öhman SG, Sundström Poromaa I, Högberg U, Sydsjö G, Skoog Svanberg A. How women perceive abortion care: A study focusing on healthy women and those with mental and posttraumatic stress. Eur J Contracept Reprod Health Care 2015; 20(3):211-22.
206. Yeung TW, Lee VC, Ng EH, Ho PC. A pilot study on the use of a 7-day course of letrozole followed by misoprostol for the termination of early pregnancy up to 63 days. Contraception 2012; 86(6):763-9.
207. Yunzal-Butler C, Sackoff J, Li W. Medication abortions among New York City residents, 2001-2008. Perspect Sex Reprod Health. 2011;43(4):218–223.
